# 

**Published:** 1887-08

**Authors:** 


					﻿JkTJG-TJST, 1887-
OLD SERIES
Vol. XXV
^I855^>-
NEW SERIES
Vol. IV.-No. 6_X/'
Mtmw
JOUBNAjj


W.F.WESTMORELAND. JO
H.VM.M1LLER. M.D.LL .D.1
JAMES A.GRAY. M.D.'
■PUBLISHED BY JAMES P.HARRISON&.C®
Atlanta,ga.
SUBSCRIPTION: $2.00 A YEAR.
				

